# Use of Cefiderocol in Management of Resistant Gram-Negative Infections in Patients Admitted to a Burn Center

**DOI:** 10.3390/microorganisms13020330

**Published:** 2025-02-03

**Authors:** Lindey C. Lane, Jonathon K. Walker, David M. Hill

**Affiliations:** Department of Pharmacy, Regional One Health, Memphis, TN 38103, USA; jwalker7@regionalonehealth.org (J.K.W.); dmhill@regionalonehealth.org (D.M.H.)

**Keywords:** burn, cefiderocol, infection, multidrug-resistant organisms

## Abstract

Cefiderocol is a novel cephalosporin antibiotic approved for urinary tract infections and hospital-acquired or ventilator-associated pneumonias caused by difficult-to-treat Gram-negative pathogens. To date, its use in treating difficult-to-treat Gram-negative infections in burn patients has been minimally described in the literature. Our aim was to evaluate cefiderocol use in a population of burn patients initiated on cefiderocol for resistant Gram-negative infections. A retrospective chart review of nine patients was conducted. Two patients were treated for pneumonia; five for bacteremia, three of which had coexisting burn wound infections or pneumonia; one was treated for a burn wound infection alone; and one patient was treated for a simultaneous burn wound infection, pneumonia, and bacteremia. The pathogens treated included the following: multidrug-resistant *Pseudomonas aeruginosa*, carbapenem-resistant *Acinetobacter baumannii*, and carbapenem-resistant *Enterobacterales*. Three isolates were confirmed as New Delhi metallo-beta-lactamase (NDM) producers, though this was likely an underestimate as genetic testing is not routinely performed at our institution and not all the isolates were tested. One of the nine patients in this study succumbed to their infection. Of note, the multidrug-resistant *Pseudomonas aeruginosa* in this patient tested intermediate to cefiderocol. The patients were treated with cefiderocol for a median of 14 days, most commonly in combination with other antimicrobial therapies. Treatment with cefiderocol appeared to be efficacious in this population of burn patients when other antibiotics routinely used for complicated infections caused by multidrug-resistant (MDR) Gram-negative pathogens showed treatment failure or resistance.

## 1. Introduction

The emergence of treatment-resistant bacteria is a growing concern and threat to public health worldwide [1]. The CDC’s *2019 Antimicrobial Threats Report* showed that nearly 3 million infections in the U.S each year are caused by antimicrobial-resistant pathogens, resulting in around 35,000 deaths [2]. Various strategies, such as better antimicrobial stewardship and prescribing practices, have been implemented at healthcare facilities to combat this growing threat, but the use of broad-spectrum antimicrobials continues to increase despite these efforts. Studies have shown this to be especially true in patients suffering burn injuries [3,4,5]. After a burn injury, patients are at an increased risk of infection due to damage to their skin, which serves as the primary barrier to infectious organisms [6,7]. This, coupled with an increased length of hospital stays, often leads to repeated and prolonged antibiotic courses for burn patients [3,6].

Cefiderocol is a novel intravenous (IV) cephalosporin antibiotic with activity against a wide range of Gram-negative bacteria (GNB). It works to penetrate the outer bacterial cell membrane both actively and passively, by acting as a siderophore and via porin channels [8]. By binding to the extracellular free iron, cefiderocol is able to use bacterial active transport channels to enter cells and primarily bind to penicillin-binding protein 3 [7]. This binding interferes with cell wall synthesis and results in cell lysis, which allows cefiderocol to overcome common Gram-negative bacterial resistance mechanisms, including porin channel mutations and efflux pump up-regulation [3]. It is also highly stable against the hydrolysis of many beta-lactamases, including serine beta-lactamases and metallo-beta-lactamases, due to its side-chain characteristics [7]. Cefiderocol is currently approved by the United States Food and Drug Administration (FDA) for use in complicated urinary tract infections (UTIs) and hospital-acquired or ventilator-associated pneumonias, but is often employed in the treatment of other types of infections caused by difficult-to-treat GNB. Its ability to overcome bacterial resistance mechanisms and its relatively safe side effect profile make cefiderocol a desirable choice for both the empiric and salvage treatment of burn patients with multidrug-resistant infections caused by GNB, particularly in combination with other agents. Despite this, its use in treating difficult-to-treat Gram-negative infections in burn patients has been minimally described in the literature. Our aim was to evaluate cefiderocol use in a population of patients admitted to a burn unit and initiated on cefiderocol for the treatment of complex infections due to resistant Gram-negative pathogens.

## 2. Materials and Methods

We conducted a retrospective case series of burn patients who received cefiderocol for the treatment of resistant Gram-negative infections in an American Burn Association (ABA)-verified burn center between 1 July 2021 and 1 May 2024. Included patients were identified by pharmacy database as recipients of cefiderocol during admission for any indication. Data were collected manually via chart review from the electronic medical records. Collected demographic information included the following: age, race, sex, and comorbid conditions. Abstracted burn injury characteristics included the following: injury mechanism, percent total body surface area burned (TBSA), percent partial- and full-thickness injury, and presence and grade of inhalation injury. Patient outcomes were collected, including total length of stay (i.e., acute index hospitalization plus associated skilled nursing or long-term care facility days when contiguous and available), mortality, and resolution of infection as indicated by repeat culture results or resolution of infectious markers and clinical improvement. Culture results, leading to initiation of cefiderocol, and susceptibilities were collected and analyzed. Cultures could have been isolated from any source, including skin swabs, tissue cultures following surgical excision, bronchoalveolar lavage (BAL), sputum, tracheal aspirate, urine, bone, or blood. This information is summarized in Table 1 and Table 2.

During the study period, our institution utilized the MALDI Biotyper (Brucker, Billerica, MA) for bacterial identification. The BD Phoenix™ (Sparks, MD) automated system was used for antimicrobial susceptibility testing. Microbial identification was based on the results of 45 chromogenic and fluorogenic substrates. Isolates of *E. coli*, *K. pneumoniae*, and *K. oxytoca* were tested for extended-spectrum beta-lactamase (ESBL) production based on differential responses to third-generation cephalosporins in the presence and absence of clavulanic acid. Resistance to meropenem or ertapenem was utilized as confirmation of carbapenem resistance, as these were the representative carbapenems on the antimicrobial susceptibility testing panel. A carbapenemase-producing Organism Detect Panel is utilized by the BD Phoenix system (Sparks, MD). Specific genetic testing for resistance at our institution requires samples to be sent out to consulting laboratories and must be requested. As such, this information was not retrospectively available for all included organisms. The rules for antibiotic reporting and interpretation of minimum inhibitory concentrations (MICs) values from the BD Phoenix™ system were based on FDA-cleared interpretations built within the automated system. All cefiderocol susceptibility was determined by utilization of Kirby–Bauer (KB) disk diffusion utilizing established Clinical and Laboratory Standards Institute breakpoints.

## 3. Results

Cefiderocol therapy was successful in eight of the nine patients (88.9%). The mean total duration of cefiderocol therapy in our study was 17.7 days, with three patients experiencing recurrent infections requiring multiple courses of cefiderocol therapy. Six of the nine patients (66.7%) were treated for bacteremia, usually secondary to a wound infection or pneumonia. All the patients received a second agent, most often amikacin, in combination with cefiderocol for at least a portion of the course. Two patients (22.2%) had culture results with a cefiderocol-resistant organism per the KB interpretation, one of which succumbed to their infection. The most common organisms treated with cefiderocol in our study were MDR *Pseudomonas* spp., CRAB, and CRE, with several patients experiencing poly-microbial infections.

### 3.1. Patient Histories

#### 3.1.1. Patient 1

A 66-year-old male with a past medical history (PMH) significant for hyperlipidemia (HLD) and hypertension (HTN) was admitted to the burn unit following a burn injury during a controlled grass fire that overtook and surrounded his tractor. He was found to have 44% TBSA partial- and full-thickness injuries to the anterior and posterior bilateral lower extremities (BLEs) and bilateral hands. He was intubated and sedated and emergent escharotomies were performed at his bedside. The initial burn wound excision (BWE) and allograft placement were performed on hospital day 1. On hospital day 5, the patient was newly febrile at 39.2 degrees Celsius and empiric vancomycin and cefepime were initiated. Blood cultures were sent and ultimately grew carbapenem-resistant *Acinetobacter baumannii* (CRAB) [Sensitive (S): amikacin (MIC 16); Resistant (R): ampicillin–sulbactam (MIC > 16/8), meropenem (MIC > 8), and cefepime (MIC > 16)]. The eravacycline KB zone of inhibition was 18 mm. Operative wound cultures were obtained following the BWE, which grew carbapenem-resistant *Enterobacter cloacae* (CRE) [S: meropenem–vaborbactam (MIC 2/8); R: amikacin (MIC 32), cefepime (MIC > 16), and meropenem (MIC > 8)] and multidrug-resistant (MDR) *Pseudomonas aeruginosa* [S: amikacin (MIC ≤ 8), ceftazidime–avibactam (MIC 4/4), and ceftolozane–tazobactam (MIC 2/4); R: cefepime (MIC 16) and meropenem (MIC 8)]. The sensitivities results were not available until post-culture day 2, at which point the antibiotics were escalated to amikacin (15 mg/kg every 36 h), high-dose ampicillin–sulbactam (9 g infused over 4 h every 8 h), and eravacycline (1 mg/kg every 12 h). The operative tissue cultures continued to be positive for carbapenem-resistant *Enterobacter* spp. On hospital day 16, a BAL grew MDR *Pseudomonas aeruginosa* (same as the previous sensitivities) and the patient developed hypotension requiring vasopressor support. The amikacin was stopped (6 days of therapy) and ceftazidime–avibactam (2.5 g every 8 h) was added for expanded coverage. The eravacycline was continued for a total of 13 days, high-dose ampicillin–sulbactam for 9 days, and ceftazidime–avibactam for 21 days, with clinical improvement and clearance of the blood cultures. The patient underwent numerous repeat BWEs and grafting procedures to close his wounds, and on hospital day 105 he developed poly-microbial pneumonia with MDR *Pseudomonas aeruginosa*, CRE, and CRAB, and bacteremia with MDR *Pseudomonas aeruginosa* (all with similar sensitivities to the prior cultures). Antibiotic therapy with amikacin and ceftazidime–avibactam was resumed. The blood cultures remained positive for MDR *Pseudomonas aeruginosa* on hospital days 106, 107, 110, and 112. The ceftazidime–avibactam was escalated to cefiderocol 1.5 g IV q8h (the estimated glomerular filtration rate declined to 40 mL/min); the amikacin was continued and Colistimethate sodium (CMS dosed at 100 mg IV q8h) was added. The blood cultures cleared on day 7 of the cefiderocol therapy and the CMS was discontinued. The cefiderocol was dose-adjusted to 750 mg IV q12h due to a decline in renal function and continued for a total of 24 days. The sensitivity to cefiderocol was never tested. On hospital day 142, the blood cultures were once again positive for MDR *Pseudomonas aeruginosa* with identical susceptibilities, and cefiderocol 1.5 g IV q8h was re-initiated. The repeat blood cultures the following day were negative. The cefiderocol therapy was continued for a total of 10 days, and the patient was discharged in stable condition to a skilled nursing facility.

#### 3.1.2. Patient 2

The patient was a 76-year-old female with a PMH significant for chronic obstructive pulmonary disease (COPD), anxiety, and depression, who underwent a laparoscopic cholecystectomy and was readmitted following the procedure for septic shock. She was found to have a bowel perforation and underwent an emergent exploratory laparotomy for a bowel resection; meropenem was initiated for *Klebsiella pneumoniae* bacteremia. The patient then developed biopsy-confirmed Ecthyma Gangrenosum and she was transferred to the burn unit from an outside hospital for wound evaluation and management. She was found to have 14% TBSA partial- and full-thickness wounds to the abdomen, genitals, and thighs. On admission, blood, urine, and wound cultures were obtained. The blood cultures were positive for MDR *Pseudomonas aeruginosa* [S: amikacin (MIC ≤ 8), ceftazidime–avibactam (MIC 8/4), and ceftolozane–tazobactam (MIC ≤ 1/4); R: aztreonam (MIC > 16), cefepime (MIC 16), and meropenem (MIC > 8)]. The admission urine culture grew Candida krusei and the wound exudate cultures were significant for MDR *Pseudomonas aeruginosa*. An antimicrobial therapy was initiated with micafungin (100 mg once daily), amikacin, and ceftolozane–tazobactam (3 g every 8 h). The repeat blood cultures were negative, and the patient was managed surgically with a wound excision and allograft application on hospital day 2. Ceftolozane–tazobactam was continued for 16 days total. Over the next few months, she underwent four additional surgical procedures and her wounds were all excised and definitively covered by hospital day 60. On hospital day 69, the patient was febrile with an up-trending procalcitonin and hypotension requiring norepinephrine and vasopressin to maintain an adequate mean arterial pressure. A bronchoscopy was performed and a specimen (BAL) was obtained with the blood cultures, and empiric vancomycin and ceftazidime–avibactam (2.5 g every 8 h) were initiated. Both the BAL and blood cultures were positive for MDR *Pseudomonas aeruginosa*, which remained sensitive to amikacin (MIC ≤ 8), but was now resistant to ceftazidime–avibactam (MIC > 8/4), ceftolozane–tazobactam (MIC > 8/4), and meropenem–vaborbactam (MIC > 16/8). Once the sensitivity results were received on hospital day 72, the ceftazidime–avibactam was then escalated to cefiderocol 2 g every 8 h and amikacin. The repeat blood cultures were negative and four days after the initiation of cefiderocol the patient was able to be weaned off vasopressor support. The cefiderocol was continued for a total of 14 days and she was discharged to a long-term acute-care facility in stable condition on hospital day 95.

#### 3.1.3. Patient 3

A 32-year-old male with no known PMH was admitted to the burn unit following a burn injury he sustained in a motor vehicle collision in which his car caught fire. On examination, he was found to have 41% TBSA deep partial- and full-thickness injuries to the face, anterior and posterior trunk, right arm, right hand, BLE, and bilateral feet, as well as a Grade I inhalation injury. The patient was then intubated and sedated, emergent escharotomies of the bilateral lower extremities were performed, and resuscitation was started. On hospital day 1, the patient was taken to the operating room (OR) for a BWE and application of an allograft. On hospital day 5, the patient was taken to the OR for a right below-the-knee amputation and a further BWE with an allograft application. Tissue cultures were obtained at this time, which grew MDR *Pseudomonas aeruginosa* [S: amikacin (MIC ≤ 8) and piperacillin–tazobactam (MIC 8/4); R: cefepime (MIC 16) and meropenem (MIC 8)]. The patient was initiated on piperacillin–tazobactam, which was continued for 9 days until the patient became febrile at 39.2 degrees Celsius and developed hypotension requiring vasopressor initiation. The blood cultures were not positive; however, repeat tissue cultures were obtained, which were persistently positive for MDR *Pseudomonas aeruginosa*. The susceptibilities to ceftazidime–avibactam and ceftolozane–tazobactam were obtained, with an MIC of 4/4 and 2/4, respectively. The piperacillin–tazobactam was escalated to ceftolozane–tazobactam 1.5g IV q8h at this time, which was continued for 11 days. The patient underwent three repeat BWEs with the application of a synthetic, biodegradable dermal substitute (BTM), and was transferred to a long-term acute-care center for continued care until the wound was well granulated and ready for autografting. He was transferred back to the burn unit on hospital day 56 with a suspicion of sepsis. A BAL was performed after respiratory desaturation and hypotension requiring vasopressors, and empiric vancomycin and ceftolozane–tazobactam were initiated. The BAL grew MDR *Pseudomonas aeruginosa*, which was later (i.e., 1 month following the patient’s discharge) determined to be a New Delhi metallo-beta-lactamase (NDM) producer, and an extended-spectrum beta-lactamase (ESBL) *Klebsiella pneumoniae*. Both isolates were sensitive to cefiderocol, and the antibiotics were changed to cefiderocol (2 g every 6 h) plus amikacin. The amikacin was continued for 2 days until clinical improvement and the cefiderocol was continued for a 7-day course. The patient was transferred back to long-term care on hospital day 67 and ultimately discharged to his home after 151 days of hospitalization.

#### 3.1.4. Patient 4

A 65-year-old female with a PMH significant for COPD and substance use disorder was admitted to the burn unit following burn injuries she sustained in a house fire. On admission, she was found to have 32.5% TBSA with partial- and full-thickness injuries to the head, neck, anterior and posterior torso, right arm, right leg, and buttocks. She was intubated for airway protection and resuscitation was initiated. The initial BWE with the placement of an allograft was performed on hospital day 2. The patient was initiated on empiric vancomycin and cefepime for suspected PNA on hospital day 3, which was de-escalated to vancomycin only after the BAL grew methicillin-sensitive *Staphylococcus aureus* (MSSA) and Streptococcus viridans. The patient underwent a second BWE and the placement of a BTM on hospital day 9, during which a deep-pass tissue culture was obtained. The cultures revealed a poly-microbial wound infection with CRAB, vancomycin-resistant Enterococcus faecium (VRE), and Corynebacterium imitans. The CRAB susceptibilities were as follows: resistant to cefepime (MIC > 16), amikacin (MIC > 32), and meropenem (MIC > 8), and intermediate to colistin (MIC 2); however, the isolate was sensitive to cefiderocol. Eravacycline was tested by disk diffusion with a KB zone of inhibition of 17 mm. The antimicrobial therapy was broadened by initiating cefiderocol (2 g every 8 h) and changing the vancomycin to linezolid. On hospital day 10 (postoperative day 1), blood cultures were sent for a new fever of 39.3 degrees Celsius, which demonstrated the growth of pan-sensitive *Pseudomonas aeruginosa*. The repeat blood cultures the following day were negative. On day 11, a BAL was performed which grew CRAB (>30 million cfu/mL) and *Pseudomonas aeruginosa* (3 million cfu/mL). The sensitivities were similar; however, for the CRAB isolate, the KB zones of inhibition to eravacycline decreased to 15 mm. The cefiderocol was continued and ceftazidime–avibactam was ultimately added for combination therapy. The patient completed 14 days of cefiderocol therapy, during which time she was able to be weaned to a trach collar, remained hemodynamically stable, and the infectious markers stabilized. She underwent two additional surgical procedures for definitive coverage with an autograft, which was completed by day 53. Ultimately, she was discharged to home on hospital day 63.

#### 3.1.5. Patient 5

A 15-year-old male was admitted to the burn unit following a flame burn injury he attained while throwing accelerant onto a trash fire, which exploded and ignited his clothes. Upon examination, he was found to have 41.5% TBSA deep and partial-thickness injuries to the bilateral upper extremity (BUE), BLE, bilateral buttocks, and the anterior and posterior torso. Blood cultures were obtained on hospital day 4, eventually demonstrating the growth of Staphylococcus epidermidis, due to an up-trending procalcitonin and new fever of 38.6 degrees Celsius, and he was initiated on vancomycin and cefepime empirically. On hospital day 5, he underwent a BWE and allograft placement, during which a post-excisional tissue culture was obtained. On hospital day 9, the tissue culture grew MDR *Pseudomonas aeruginosa* [S: amikacin (MIC ≤ 8); R: aztreonam (MIC > 16), cefepime (MIC > 16), meropenem (MIC > 8), piperacillin–tazobactam (MIC > 64/4), ceftazidime–avibactam (MIC > 8/4), and ceftolozane–tazobactam (MIC > 8/4)]. The cefepime was subsequently changed to amikacin and cefiderocol (2 g every 8 h), and the vancomycin was continued for Gram-positive bacteremia. The repeat blood cultures remained negative and the patient completed a 7-day course of vancomycin therapy. The patient underwent continued BWEs on hospital days 10 and 14, where the tissue cultures remained positive for MDR *Pseudomonas aeruginosa*. The patient continued to be febrile; however, procalcitonin continued to downtrend, and the patient remained hemodynamically stable. No repeat tissue cultures were obtained and the patient completed a 9-day course of amikacin on hospital day 18. The cefiderocol was continued until hospital day 25, for a total of 17 days of therapy. On hospital day 72, the patient was ultimately discharged to home, with complete wound closure after seven surgical procedures.

#### 3.1.6. Patient 6

The patient was a 65-year-old female, with a PMH significant for COPD on home oxygen, chronic kidney disease (CKD), HTN, anxiety, and depression, admitted to the burn unit following burns she sustained in a house fire. Upon evaluation, she was noted to have 20% TBSA deep partial- and full-thickness injuries to the anterior and posterior torso, right arm, right buttock, and left thigh. An initial BWE was performed on hospital day 2 with the application of an allograft. Three days after admission the patient developed an *Escherichia coli* urinary tract infection (UTI) and methicillin-resistant *Staphylococcus aureus* (MRSA) pneumonia, for which vancomycin and cefepime were initiated. The patient returned to the OR on hospital day 7 for an additional BWE with the placement of an autograft and dermal substitute, which was well tolerated, but she returned to the unit intubated and sedated. She appeared septic on hospital day 14, with a new fever of 38.3 degrees Celsius, rising procalcitonin, and significant non-volume-responsive hypotension. Vasopressor support with norepinephrine was initiated and blood cultures were obtained. The cefepime was empirically escalated to ceftazidime–avibactam, and the vancomycin was continued. Continuous venovenous hemofiltration (CVVH) was initiated on hospital day 16, following progressively worsening renal function with lactic acidosis, and vasopressin was added due to increasing norepinephrine requirements. Blood and BAL cultures were obtained, revealing MDR *Pseudomonas aeruginosa* [S: amikacin (MIC 16) and aztreonam (MIC ≤ 2); R: cefepime (MIC > 16), ceftazidime–avibactam (MIC > 8/4), ceftolozane–tazobactam (MIC > 8/4), meropenem (MIC > 8), and piperacillin–tazobactam (MIC > 64/4)], bacteremia, and pneumonia. The cefepime therapy was then escalated to cefiderocol 1.5 g IV q8h (dosing based on CVVH effluent rate of 4 L/hr) and amikacin. The cefiderocol susceptibility was subsequently determined to be intermediate. The vancomycin was discontinued after the completion of a 14-day course. The patient returned to the OR on hospital day 16 for a repeat BWE and application of an allograft and BTM. The operative tissue cultures were also positive for MDR *Pseudomonas aeruginosa*, which was sent to an outside laboratory and confirmation was returned 1 month later that it was an NDM producer. A repeat BWE and grafting were performed on hospital day 24, with tissue culture obtainment. The cultures continued to be positive for MDR *Pseudomonas aeruginosa* and were now also growing VRE. The vancomycin was changed to linezolid for VRE coverage. The patient continued to require vasopressor support and CVVH. On hospital day 28, the patient was transitioned to comfort measures and ultimately succumbed to her injuries.

#### 3.1.7. Patient 7

The patient was a 41-year-old female with a PMH of depression, anxiety, schizophrenia, and substance use disorder, who was admitted to the burn unit for flame burns sustained in a house fire. She arrived at the unit intubated and sedated. On examination, the patient was found to have 38% TBSA partial- and full-thickness burns to the face, neck, buttocks, anterior and posterior trunk, BLE, BUE, and a Grade III inhalation injury. On hospital day 2, the patient developed non-volume-responsive hypotension and was initiated on vasopressor support and empiric vancomycin and cefepime. She underwent her first BWE with an allograft placement on hospital day 4. She returned to the OR on hospital day 11 for a subsequent BWE and placement of a dermal substitute, and deep-pass tissue cultures were obtained. The operative cultures results on hospital day 13 revealed a poly-microbial infection, including CRAB [R: amikacin (MIC > 32), cefepime (MIC > 16), meropenem (MIC > 8), and ampicillin–sulbactam (MIC 16/8)], and the cefepime was escalated to high-dose ampicillin–sulbactam, meropenem, and amikacin. Additional sensitivity testing results on hospital day 15 revealed the isolate was sensitive to cefiderocol (KB 19 mm) and eravacycline (KB 16 mm), and the antibiotics were subsequently changed to eravacycline and cefiderocol 2 g IV q8h. The eravacycline was discontinued on hospital day 17 after confirmation of cefiderocol sensitivity, and the cefiderocol was continued for 28 days. The patient underwent eight additional surgical procedures and operative wound cultures were obtained, which grew CRAB, VRE, MRSA, CRE, Stenotrophomonas maltophilia [S: sulfamethoxazole–trimethoprim (MIC ≤ 0.5/9.5)], and MDR Achromobacter xylosoxidans [S: piperacillin–tazobactam (MIC ≤ 2/4) and sulfamethoxazole–trimethoprim (MIC ≤ 0.5/9.5); R: amikacin (MIC > 32), aztreonam (MIC > 16), and cefepime (MIC > 16)]. On hospital day 30, the patient developed bacteremia with MDR Achromobacter xylosoxidans [cefiderocol KB 25 mm, ceftazidime–avibactam KB 22 mm, and ceftolozane–tazobactam KB 6 mm] and CRAB [S: cefiderocol; ceftazidime–avibactam KB 6 mm, ceftolozane–tazobactam KB 6 mm, and eravacycline KB 18 mm]. The cefiderocol was continued and ceftazidime–avibactam was added at this time. The blood cultures remained positive on hospital days 31, 32, 34, 41, 49, and 51, likely representing an inadequate source control. Eravacycline, cefiderocol, imipenem–cilastatin, amikacin, high-dose ampicillin–sulbactam, and ceftazidime–avibactam were used in combination until the blood cultures cleared on hospital day 52. The cefiderocol was continued for a total of 35 days throughout hospitalization, with resistance to cefiderocol noted for the CRAB isolate on the final blood cultures. All the wounds were definitively covered by hospital day 68 and the patient was transferred to a sub-acute facility one month later.

#### 3.1.8. Patient 8

The patient was a 42-year-old male with no significant PMH admitted to the burn unit for flame burns sustained in a house fire. He arrived at the unit intubated and sedated on norepinephrine for hypotension following cardiac arrest sustained en route. On examination, he was found to have 55% TBSA partial- and full-thickness burns to the head, neck, anterior and posterior torso, BUE, BLE, and a Grade II inhalation injury. Following resuscitation, the patient developed an acute kidney injury with rhabdomyolysis and lactic acidosis, which required the initiation of CVVH on hospital day 3. Empiric vancomycin and cefepime were initiated for suspected sepsis. The only positive cultures were an admission urine culture with pan-sensitive *Klebsiella pneumoniae* and *Escherichia coli*. The patient underwent BWEs and allografts on hospital days 4 and 7, at which time a tissue culture was obtained that grew *Enterobacter hormaechei* [S: amikacin (MIC ≤ 8), cefepime (MIC ≤ 1), ertapenem (MIC ≤ 0.25), meropenem (MIC ≤ 0.5), and piperacillin–tazobactam (MIC ≤ 4/4); R: ampicillin–sulbactam (MIC 16/8) and aztreonam (MIC > 16)]. As a result, the cefepime was continued and the vancomycin discontinued. A subsequent BWE and allograft placement occurred on hospital day 11 with a tissue culture, which remained positive for *Enterobacter hormaechei*. On hospital day 14, the patient was transitioned from CVVH to intermittent hemodialysis. The sensitivities results on hospital day 17, however, showed that the *Enterobacter hormaechei* isolate was now carbapenem-resistant [R: meropenem (MIC 8) and cefepime (MIC > 8)]. It was susceptible to meropenem–vaborbactam (MIC ≤ 2/8). The blood cultures obtained 2 days later were also positive for carbapenem-resistant *Enterobacter hormaechei* and MRSA. The antibiotic therapy was empirically changed to ceftazidime–avibactam and daptomycin until the CRE was determined to be resistant to ceftazidime–avibactam (MIC > 8/4) 3 days later. The isolate was also now resistant to ceftolozane–tazobactam (MIC > 8/4) and meropenem–vaborbactam (MIC > 16/8), but susceptible to cefiderocol, which was initiated as a renally adjusted regimen (750 mg IV q12h). The repeat blood cultures obtained 3 days later were negative. Also, on hospital day 17, the patient returned to the OR for a BWE and application of a BTM with the obtainment of a tissue culture. The culture remained positive for carbapenem-resistant *Enterobacter hormaechei* (identical susceptibilities as previously) as well as MDR *Pseudomonas aeruginosa* (S: cefiderocol). On hospital day 19, the patient underwent a BWE and placement of an autograft and BTM. No repeat wound cultures were obtained following source control and wound coverage; however, the blood cultures remained negative. The cefiderocol was continued for a total of 16 days. All the wounds were definitively covered by hospital day 50, and the patient was discharged to our burn-specific inpatient rehabilitation hospital on hospital day 66.

#### 3.1.9. Patient 9

A 39-year-old male with a PMH significant for hepatitis C and substance use disorder was admitted to the burn unit following self-immolation with an accelerant. On arrival, he was found to have 57% TBSA full-thickness injuries to the face, head, neck, BUE, bilateral hands, bilateral thighs, and anterior and posterior torso. Emergent escharotomies were performed at his bedside. On hospital day 2, the patient was taken for his first BWE and application of an allograft. On hospital day 6, the patient was taken for a BWE of the remaining unexcised areas with the application of an allograft, and operative tissue cultures were obtained. Postoperatively, the patient was febrile with up-trending procalcitonin and non-volume-responsive hypotension, requiring the initiation of vasopressor support. Empiric vancomycin and cefepime were started at this time. The operative culture was positive for MDR *Pseudomonas aeruginosa* [S: amikacin (MIC ≤ 8), aztreonam (MIC 8), cefepime (MIC 8), ceftazidime–avibactam (MIC 2/4), and ceftolozane–tazobactam (MIC ≤ 1/4); R: piperacillin–tazobactam (MIC 64/4) and meropenem (MIC 8)]. When the sensitivity results were returned on day 10, the cefepime was then escalated to ceftolozane–tazobactam. A repeat BWE with the application of an autograft was completed on hospital day 11, and operative tissue cultures were obtained, which ultimately grew VRE. Daptomycin was added when the sensitivities results were returned on day 16. The blood cultures obtained on hospital day 19 were positive for *Enterobacter hormaechei* [S: amikacin (MIC ≤ 8), meropenem (MIC ≤ 0.5), ceftazidime–avibactam (MIC 1/4), and cefiderocol; R: aztreonam (MIC > 16), cefepime (MIC > 16), piperacillin–tazobactam (MIC > 64/4), and ceftolozane–tazobactam (MIC > 8/4)] and Acinetobacter nosocomialis, which was pan-sensitive. The repeat blood cultures remained positive on hospital days 20, 21, and 24; however, the Enterobacter isolate was now resistant to meropenem (MIC > 8), ceftazidime–avibactam (MIC > 8/4), and meropenem–vaborbactam (MIC > 16/8). The antibiotics were escalated to amikacin and cefiderocol 2g IV q6h for 2 days, when the infectious disease service was consulted and the antibiotics were changed to meropenem–vaborbactam and aztreonam. The CRE isolate was sent to an outside laboratory and was confirmed to be an NDM producer one month later. The blood cultures cleared on hospital day 25 and the meropenem–vaborbactam and aztreonam were continued for a total of 18 days. A repeat BWE and application of an autograft were completed on hospital day 38. Postoperatively, the patient remained febrile, though hemodynamically stable, and blood cultures were obtained. The cultures were positive for VRE and MDR *Pseudomonas aeruginosa* [S: amikacin (MIC ≤ 8), ceftazidime–avibactam (MIC ≤ 0.25/4), ceftolozane–tazobactam (MIC ≤ 1/4), and cefiderocol; R: aztreonam (MIC 16) and meropenem (MIC 8)]. A combination therapy with cefiderocol 2g IV q8h, amikacin, and daptomycin was initiated on hospital day 44, and the blood cultures cleared the following day. The remaining non-closed wounds were suspected to be the source of infection, as time was required for the donor sites maturation for reharvesting. By hospital day 47, repeat blood cultures were again positive for MDR *Pseudomonas aeruginosa* and *Enterobacter hormaechei*, which were now resistant to ceftazidime–avibactam, but remained sensitive to cefiderocol. The repeat cultures on hospital day 48 were negative and remained negative throughout the patient’s stay. Cefiderocol, amikacin, and daptomycin were continued for a total course of 10 days. All the burn wounds were definitively covered by hospital day 81, and the patient was discharged to home on hospital day 102.

## 4. Discussion

Antimicrobial resistance is a significant problem in burn centers worldwide and the therapeutic options for managing these infections are limited. Cefiderocol is a last-line agent for many resistant Gram-negative infections, though its effectiveness in burn patients has not been well studied. This study assessed the clinical efficacy of cefiderocol in nine patients admitted to a burn unit for the treatment of resistant Gram-negative infections. Eight of the nine patients (88.9%) sustained flame injuries (three with concomitant inhalation injuries), while one patient was transferred from an outside facility due to complicated ecthyma–gangrenosum. This is the first study detailing the clinical course and outcomes associated with cefiderocol use in a burn center.

Patients with complex wounds, particularly patients with a burn injury, have been understudied in phase 3 studies [8]. However, this patient population has been shown to be at high risk for the development of infections with multidrug-resistant organisms. Following a burn injury, patients lose their primary barrier to infection and experience complex and protracted hospital courses due to limited donor sites for autografting and the closure of wounds that often need to be given time to heal and reharvest for additional coverage. During this prolonged period, patients are exposed to many pathogens and likely many courses of antimicrobial therapy, contributing to an increased incidence of these resistant infections [6,9,10]. The prevalence of multidrug-resistant organisms in burn centers varies, but the reported incidence has been shown to be as high as 80% [11,12,13]. This fact lends support to the importance of studying the effectiveness of cefiderocol in this population.

Following a burn injury, patients have significant alterations to the pharmacokinetic parameters related to drug dosing, and may require higher doses even for isolates with MICs deemed susceptible. Many factors play a role in these pharmacokinetic changes, including the TBSA and burn depth, age, renal function, serum protein concentrations, and time from injury. The initial phase after a burn injury is characterized by systemic vasodilation, a leaky vasculature, and decreased renal perfusion leading to reduced drug clearance. Additionally, blood is shunted away from tissues such as the skin at this time, which may impact antimicrobial penetration to the site of infection. Following the initial 48 h, assuming appropriate resuscitative efforts, a hypermetabolic state with increased renal perfusion and augmented clearance of many antimicrobials is noted, and may persist for years after injury. This, coupled with the potential increased drug clearance from the exudate leakage from the burn wounds may necessitate escalated dosing strategies. On the other hand, a burn injury has been shown to lead to high rates of acute kidney injury and the initiation of renal replacement therapy [14]. Cefiderocol dose-adjustment recommendations based on the dialysis mode and effluent rates are available; however, these recommendations were developed from burn injury-limited phase 3 data, which included 16 patients receiving renal replacement therapy [8,15]. There are no data analyzing the depth of injury (i.e., degree of burn) as an adjustment factor for cefiderocol dosing. Few studies have evaluated the ability of cefiderocol to penetrate to the site of infection (i.e., skin/subcutaneous tissue) in burn patients. However, a study by Mueller et al. confirmed the ability of cefiderocol to penetrate in adequate concentrations to treat an extensively drug-resistant *Pseudomonas aeruginosa* in a patient with Pyoderma gangrenosum, a rare dermatologic disorder [16]. Another study conducted in healthy volunteers determined that cefiderocol reached sufficient concentrations in the soft tissues for the management of skin and soft-tissue infections [17]. Cefiderocol dosing has not been well established in this subset of patients and requires further exploration to determine the optimal dosing of patients with severe burn injury.

The Infectious Diseases Society of America (IDSA) currently suggests cefiderocol may be used as treatment for CRE, CRAB, and MDR *Pseudomonas* spp., typically as an alternative, combination, or salvage therapy. Despite these recommendations, cefiderocol susceptibility testing is not available on any automated antimicrobial susceptibility testing system, and requires either broth dilution or disk diffusion testing with iron-depleted media to ensure accurate results. As such, the sensitivity results to cefiderocol are often delayed by 3–4 days once a resistant organism is identified following automated sensitivity testing. The resources do not exist for every institution to perform routine testing. When performed, a discrepancy exists in the established breakpoints for cefiderocol sensitivity testing between regulatory organizations, such as the Clinical and Laboratory Standards Institute (CLSI) and the Food and Drug Administration (FDA) [18]. These differences make establishing the breakpoint cut-offs for isolate susceptibility difficult. Liability concerns may also lessen institutions’ willingness to perform additional testing without clear standards. This is especially true for isolates suspected or confirmed to be New Delhi metallo-beta-lactamase (NDM) producers. Studies have highlighted the differences in the percent susceptibility of the species within the order Enterobacterales confirmed to be NDM producers, with 84% of the isolates regarded as sensitive when utilizing the CLSI established breakpoints as compared to 51% of the isolates when using the breakpoints determined by the FDA [18]. Three isolates in this study were confirmed NDM producers; however, this testing is not routinely performed at our institution and not all isolates were tested. NDM production is tested as a send out, and the average time for a report on this genetic testing was around 1 month after receipt. Cefiderocol is one of only two preferred treatment strategies for NDM-producing organisms per the IDSA guidelines for the management of resistant Gram-negative infections, which makes it particularly useful for empiric use when NDM production is suspected [19]. Sensitivity discrepancies in NDM-producing organisms highlights the importance of making the genetic testing of these isolates commonplace, as these isolates may require alternative therapies or combination therapy with other extended-spectrum antimicrobials.

The adverse effect profile of cefiderocol is similar to that of other broad-spectrum antimicrobials, and is commonly associated with mild side effects, including nausea, vomiting, and diarrhea. However, the most common side effects include hypokalemia and elevated liver enzymes. Similar to other beta-lactam antimicrobials, potentially severe side effects include seizures and Clostridioides difficile infection. One patient in our study developed a Clostridioides difficile infection; however, this patient had been exposed to numerous broad-spectrum antimicrobials and thus this could not be directly attributed to cefiderocol use.

## 5. Conclusions

Our retrospective observation of nine burn patients receiving cefiderocol for resistant Gram-negative infections found it to be an effective option in this specialized patient population, including those with bacteremia, when traditional agents were ineffective. More high-quality data are needed to validate the utility of cefiderocol in this patient population. Future research should focus on optimizing the pharmacodynamics of cefiderocol through a pharmacokinetic analysis of patients following burn and multitrauma injury, and publishing the outcomes from a multicenter sample, as treatment courses and resistance profiles vary between centers.

## Figures and Tables

**Table 1:** Patient demographics and injury characteristics.

Patient No.	Age (Years)	Race	Sex	Comorbidities	Injury Mechanism	TBSA	Partial Thickness	Full Thickness
1	67	AA	M	HTN, HLD	Flame	44%	6%	38%
2	76	White	F	COPD, GERD, Depression, Anxiety, Osteoarthritis	Ecthyma Gangrenosum	14%	0%	14%
3	32	AA	M	None	Flame/Inhalation	41%	3.5%	37.5%
4	65	White	F	None	Flame	32.5%	19%	13.5%
5	15	AA	M	ADHD	Flame	41.5%	13.5%	23%
6	66	White	F	COPD, HTN, Anxiety, Depression	Flame	20%	8.5%	11.5%
7	42	White	F	Depression, Anxiety, Schizophrenia, SUD	Flame/Inhalation	31.5%	19%	12.5%
8	42	White	M	None	Flame/Inhalation	55%	10.5%	44.5%
9	41	White	M	Hepatitis C, SUD	Flame	57%	5%	52%

Abbreviations: AA, African American; ADHD, attention-deficit hyperactivity disorder; COPD, chronic obstructive pulmonary disease; F, female; GERD, Gastroesophageal Reflux Disease; HTN, hypertension; HLD, hyperlipidemia; M, male; No., number; SUD, substance use disorder; TBSA, total body surface area burned.

**Table 2:** Infection characteristics and outcomes.

Patient No.	Culture Specimens	Infection Source	Pathogen	Total Cefiderocol Duration	Prior Antimicrobial Therapy	Combination Therapy with Cefiderocol	Mortality
1	Wound exudate, Blood	Bacteremia	CRE, MDR PSA	34 days	VAN, CFP, MER, CTZ-AVI, CZN-TAZ, ERA, AMI, AMP-SUL, CMS	AMI, CMS, VAN	No
2	Blood, BAL	PNA, Bacteremia	MDR PSA	14 days	AMI, CZN-TAZ, CTZ-AVI, LIN, MER, MICA, VAN	AMI, CFZ	No
3	BAL	PNA	MDR PSA	7 days	VAN, CZN-TAZ	AMI	No
4	BAL	PNA	CRAB	14 days	AMP-SUL, CFP, VAN	AMI, LIN	No
5	Tissue	Wound	MDR PSA	17 days	CFP, VAN	AMI	No
6	Blood, BAL, Tissue	PNA, Bacteremia, Wound	MDR PSA	10 days	AMI, CFP, CTZ-AVI, VAN	AMI	Yes
7	Tissue, Blood	Wound, Bacteremia	CRAB, CRE, PSA, VRE, *Achromob-acter*	35 days	AMP-SUL, AMI, CFP, MER, VAN	CTZ-AVI, DPT, ERA, SUF-TRP	No
8	Tissue, Blood	Wound, Bacteremia	CRE	16 days	CFP, CTZ-AVI, DPT	AMI, AZT, VAN	No
9	Blood	Bacteremia	CRE, MDR PSA, VRE	12 days	AMI, CFP, CZN-TAZ, DPT, VAN, MER-VBR, AZT	AMI, DPT	No

Abbreviations: AMI, amikacin; AMP-SUL, ampicillin–sulbactam; AZT, aztreonam; BAL, bronchoalveolar lavage; CFZ, cefazolin; CFP, cefepime; CMS, colistimethate sodium; CRAB, carbapenem-resistant *Acinetobacter buamannii*; CRE, carbapenem-resistant *Enterobacterales*; CTZ-AVI, ceftazidime–avibactam; CZN-TAZ, ceftolozane–tazobactam; DPT, daptomycin; ERA, eravacycline; LIN, linezolid; MER, meropenem; MER-VBR, meropenem–vaborbactam; MICA, micafungin; MDR PSA, multidrug-resistant *Pseudomonas aeruginosa*; No., number; PNA, pneumonia; SUF-TRP, sulfamethoxazole–trimethoprim; VAN, vancomycin; VRE, Vancomycin-Resistant Enterococcus.

## Data Availability

The dataset is available on request from the authors.
